# An outbreak of coxsackievirus A6 hand, foot, and mouth disease associated with onychomadesis in Taiwan, 2010

**DOI:** 10.1186/1471-2334-11-346

**Published:** 2011-12-14

**Authors:** Sung-Hsi Wei, Yuan-Pin Huang, Ming-Chih Liu, Tsung-Pei Tsou, Hui-Chen Lin, Tsuey-Li Lin, Chen-Yen Tsai, Yen-Nan Chao, Luan-Yin Chang, Chun-Ming Hsu

**Affiliations:** 1 Taiwan Centers for Disease Control, Taipei, Taiwan; 2 Institute of Public Health, National Yang-Ming University, Taipei, Taiwan; 3 Department of Pediatrics, China Medical University Beigang Hospital, Yunlin, Taiwan; 4 Department of Pediatrics, Kuang Tien General Hospital, Taichung, Taiwan; 5 Division of Infectious Diseases, Department of Pediatrics, National Taiwan University Hospital, College of Medicine, National Taiwan University, No. 7, Chung-Shan South Road, Taipei 100, Taiwan

**Keywords:** Coxsackievirus A6, Hand, foot, and mouth disease, Onychomadesis

## Abstract

**Background:**

In 2010, an outbreak of coxsackievirus A6 (CA6) hand, foot and mouth disease (HFMD) occurred in Taiwan and some patients presented with onychomadesis and desquamation following HFMD. Therefore, we performed an epidemiological and molecular investigation to elucidate the characteristics of this outbreak.

**Methods:**

Patients who had HFMD with positive enterovirus isolation results were enrolled. We performed a telephone interview with enrolled patients or their caregivers to collect information concerning symptoms, treatments, the presence of desquamation, and the presence of nail abnormalities. The serotypes of the enterovirus isolates were determined using indirect immunofluorescence assays. The VP1 gene was sequenced and the phylogenetic tree for the current CA6 strains in 2010, 52 previous CA6 strains isolated in Taiwan from 1998 through 2009, along with 8 reference sequences from other countries was constructed using the neighbor-joining command in MEGA software.

**Results:**

Of the 130 patients with laboratory-confirmed CA6 infection, some patients with CA6 infection also had eruptions around the perioral area (28, 22%), the trunk and/or the neck (39, 30%) and generalized skin eruptions (6, 5%) in addition to the typical presentation of skin eruptions on the hands, feet, and mouths. Sixty-six (51%) CA6 patients experienced desquamation of palms and soles after the infection episode and 48 (37%) CA6 patients developed onychomadesis, which only occurred in 7 (5%) of 145 cases with non-CA6 enterovirus infection (*p *< 0.001). The sequences of viral protein 1 of CA6 in 2010 differ from those found in Taiwan before 2010, but are similar to those found in patients in Finland in 2008.

**Conclusions:**

HFMD patients with CA6 infection experienced symptoms targeting a broader spectrum of skin sites and more profound tissue destruction, i.e., desquamation and nail abnormalities.

## Background

Since 2000, there have been sporadic reports of associations between hand, foot and mouth disease (HFMD) and occurrences of onychomadesis [1-9]. Onychomadesis results from nail matrix arrest and both fingernails and toenails may be involved. The nail abnormalities associated with HFMD manifest with Beau's line, defined as transverse ridging of the nail plate, or onychomadesis, defined as nail separation from the nail matrix, which may extend distally to the proximal portion of the nail bed. Despite reports in the literature, however, evidence of the associations between HFMD and nail abnormalities remains limited.

Following a major enterovirus 71 (EV71) epidemic in 1998, a virology reference laboratories network was established by the Taiwan Centers for Disease Control (TCDC) to monitor the trends of viral infections in Taiwan [10,11]. In 2010, ten clinical virology laboratories were qualified to be regional virology reference laboratories by the TCDC. Physicians in public health centers, clinics or hospitals were eligible to submit clinical samples of patients with respiratory tract infection or enterovirus infection to contracted laboratories nearby for laboratory diagnosis. For each specimen from a patient with suspected enterovirus infection, virus isolation and real-time reverse transcriptase-polymerase chain reaction (RT-PCR) specific for enterovirus type 71 was performed in a contracted laboratory. In addition to notifying the clinical physicians, the test results were stored and maintained in a digital network database. The virus isolates were transported to and preserved in the TCDC's central biological materials bank.

**Figure 1:**
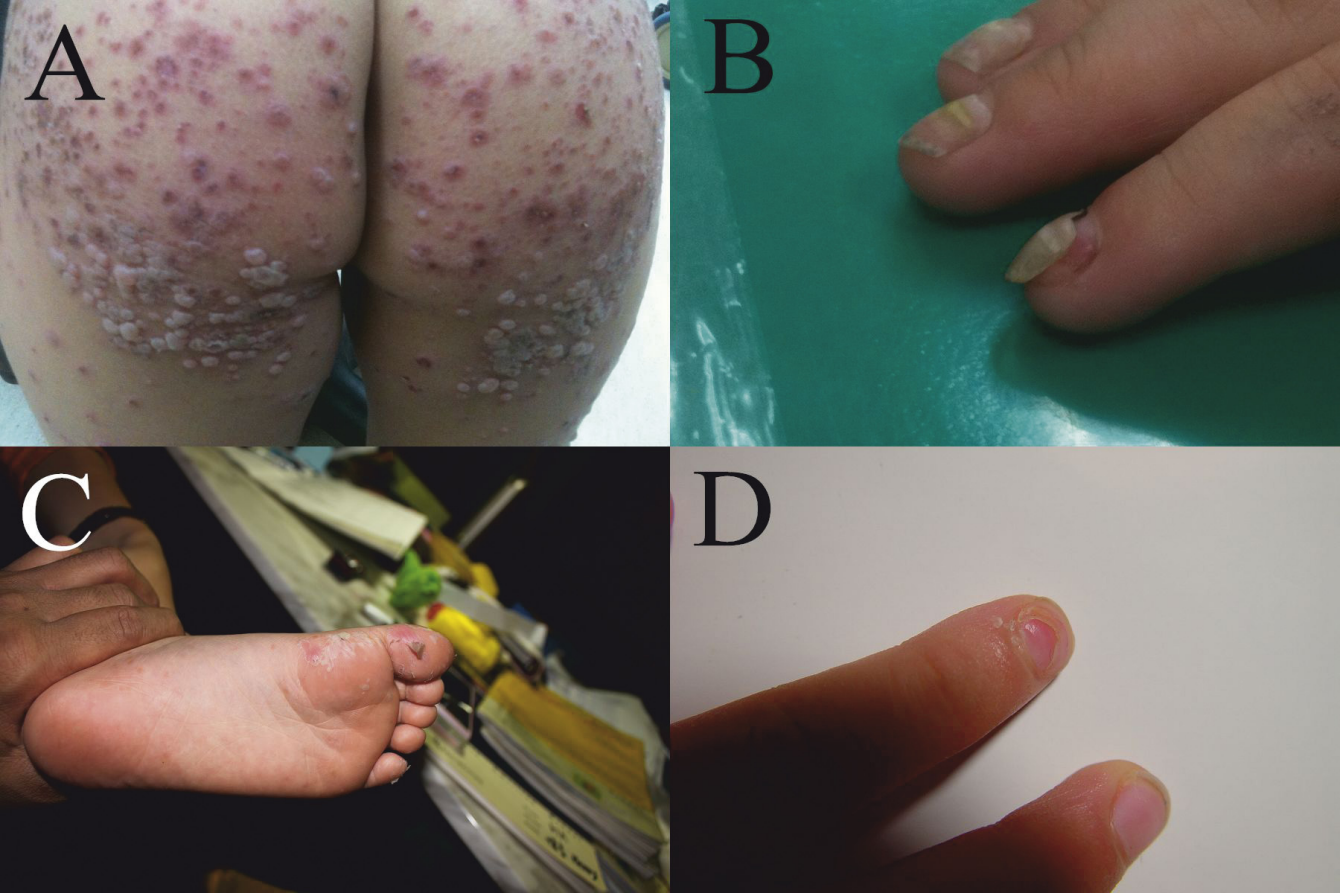
**A 2-year-5-month-old boy experienced prominent skin eruptions and vesicles on his buttocks (**a**)**. A 2-year-6-month-old boy had fingernail abnormalities 3 weeks after hand, foot, and mouth disease (**b**). Another 4-year-old boy presented with desquamation on his feet 1 week after the CA6 infection episode (**c**); he developed nail shedding on his right index finger in the following weeks (**d**) and recovered gradually.

In 2010, physicians in Taiwan noticed an outbreak of onychomadesis. Most of the involved patients had HFMD prior to the onset of the nail abnormalities (Figure 1). The patients with HFMD also presented with skin eruptions at unusual skin sites. Therefore, we performed an epidemiological and molecular investigation to elucidate the characteristics of this outbreak.

## Methods

Patients diagnosed with HFMD with positive enterovirus isolation results in the virology reference laboratories network database from July through December, 2010 were eligible for analysis in the study. Demographic data, including gender and age, and the virus isolation information were obtained from the virology reference laboratories network database. After obtaining approval from the Institutional Review Board of the TCDC and informed consent for participation in the study from the patients or their guardians, we performed a telephone interview with eligible patients or their caregivers to collect information concerning symptoms, treatments, the presence of desquamation within 2 weeks of the HFMD episode, and the presence of nail abnormalities within 8 weeks of the HFMD episode. Nail abnormalities included Beau's line, defined as transverse ridging of the nail plate, or onychomadesis, defined as nail separation starting at the matrix, which may extend distally to the proximal portion of the nail bed.

The serotypes of the enterovirus isolates were determined using an in-house indirect immunofluorescence assay (IFA) kit [12] and a commercial IFA kit (Chemicon International, Temecula, CA, USA). For the sequencing of the VP1 (viral protein 1) gene, viral RNA was extracted using a QIAamp Viral RNA Mini Kit (Qiagen, Santa Clara, CA). One-step RT-PCR of the VP1 gene was performed as described previously [13]. The products were confirmed by agarose electrophoresis and were then sequenced using a BigDye Terminator Ready Reaction Cycle Sequencing Kit and an automated sequencer ABI 3730 (Applied Biosystems, Foster City, CA, USA). Obtained sequences were compared with reference EV sequences in GenBank using BLAST [14] and confirmed by phylogenetic analysis. All sequences were prepared and aligned by BioEdit (version 7.0.9.0) with the Clustal W program [15]. The phylogenetic tree for the current CA6 strains in 2010, 52 previous CA6 strains isolated in Taiwan from 1998 through 2009, along with 8 reference sequences from other countries was constructed using the neighbor-joining command in MEGA software (version 4) in order to perform bootstrap analysis with 1,000 replications [16].

Stata software, version 10 (Stata Corp LP, College Station, Texas, USA), was used for statistical analyses. The Fisher's exact test or chi-square test was used to compare categorical variables, and the Mann-Whitney *U *test was used to compare continuous variables. A *p *value of less than 0.05 was considered significant.

## Results

From July through December of 2010, a total of 398 patients diagnosed with HFMD with positive enterovirus isolation results in the virology reference laboratories database were selected. Among the 398 patients, 275 patients (69%) completed the telephone interview and were enrolled in the analysis. Enterovirus was isolated from the pharyngeal swabs of 274 patients and the rectal swab of 1 patient. Of the 275 patients, 252 (92%) of the patients' specimens were submitted within 3 days of clinical presentation (median duration, 1 day; range, 0 to 8 days).

CA6 was isolated from the clinical specimens in 130 patients. The enterovirus types of the other 145 patients included CA16 (110; 76%), CA5 (13; 9%), CA4 (5; 3%), EV71 (4; 3%), CA2 (3; 2%), CB5 (3; 2%), echovirus 4 (3; 2%), non-typable enterovirus (3; 2%) and echovirus 30 (1; 1%). Table 1 shows the demographic information and clinical symptoms of patients infected with CA6 and non-CA6 enterovirus. No significant differences existed between the CA6 and non-CA6 groups in terms of age and gender distribution. Patients that contracted CA6 were more frequently hospitalized, but none of these patients were treated in an intensive care unit. All the 275 patients recovered without complications.

**Table 1:** Demographic information and clinical manifestations of patients with hand, foot, and mouth disease from July through December 2010

	CA6 infection (N = 130)	Non-CA6 infection (N = 145)	P*
Median age in years (range)	2 (0-44)	3 (0-13)	0.16

Male/female	81/49	89/56	0.90

Hospitalization	70 (54%)	59 (41%)	0.03

Intensive care unit stay	0	2 (1%)	0.50

Fever	106 (82%)	121 (83%)	0.87

Eruption around the oral area	28 (22%)	7 (5%)	<0.001

Eruption at the trunk and/or neck	39 (30%)	24 (17%)	0.01

Desquamation	66 (51%)	28 (19%)	<0.001

Nail abnormalities**	48 (37%)	7 (5%)	<0.001

*P values were measured using the Mann-Whitney *U *test for age, Fisher's exact test for intensive care unit stay, and chi-square test for the other parameters. CA6 denotes coxsackievirus A6. **Nail abnormalities included Beau's line, defined as transverse ridging of the nail plate, or onychomadesis, defined as nail separation starting at the matrix, which may extend distally to the proximal portion of the nail bed.

In addition to the typical presentations of skin eruptions on the hands, feet and mouth, some patients with CA6 infection also had eruptions around the perioral area (28, 22%), the trunk and/or the neck (39, 30%). Six (5%) patients with CA6 infection experienced generalized skin eruptions. Sixty-six (51%) patients with CA 6 infection experienced desquamation of the palms and soles after the infection episode. Nail abnormalities were found in 48 (37%) of the 130 patients with CA6 infection. All 48 patients denied a past history of major systemic disease or nail trauma in the 8 weeks prior to the onset of the nail abnormalities. Among the 48 patients with nail abnormalities, 33 (69%) also had desquamation before or during the presentation of the nail abnormalities; presentation of nail abnormalities was significantly associated with the presentation of desquamation (*p *= 0.002). The median age of the 48 patients with nail abnormalities was 2 years old (range, 0-34 years). Among the 48 patients, 34 (71%) remembered the details of nail abnormalities. The average number of involved digits was six (range, 1-20) and the most common involved digit was the right thumb (24/34, 71%). Only 7 (5%) of 145 cases with non-CA6 enterovirus infection, including 4 cases caused by CA 16, 2 cases caused by EV 71, and 1 case caused by echovirus 30, experienced nail abnormalities after the HFMD episode. CA6 infection was significantly associated with developing nail abnormalities (*p *< 0.001).

**Figure 2:**
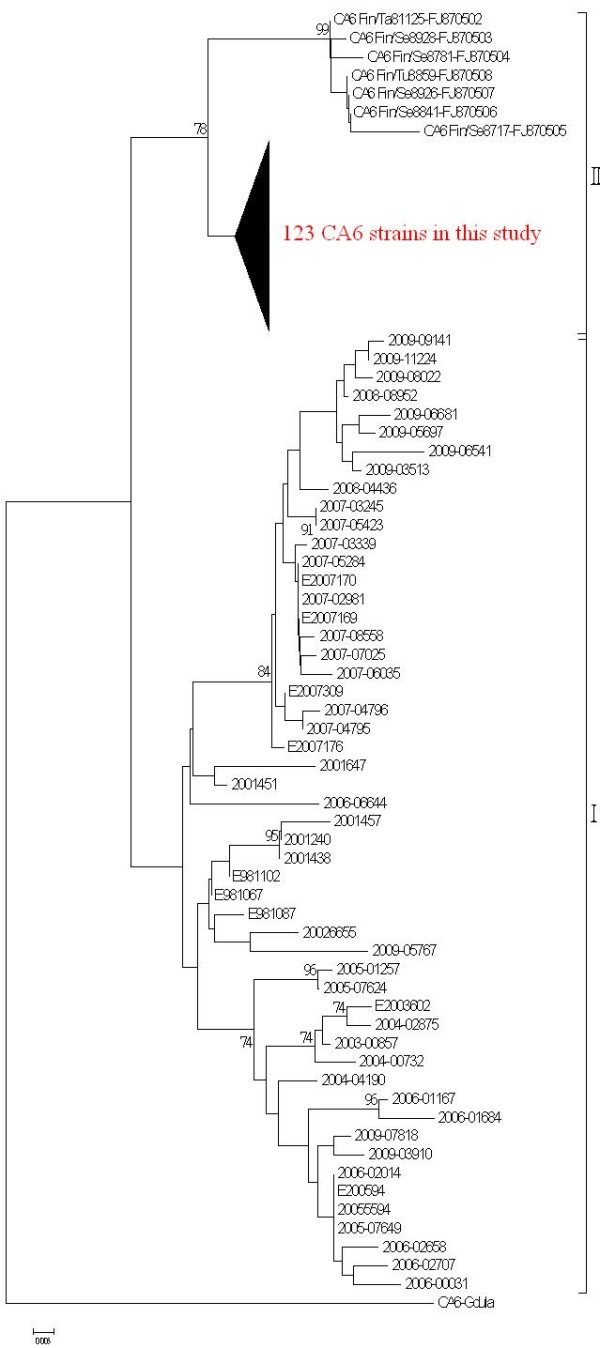
**Phylogenetic analysis of Coxsackievirus A6 strains based on the partial VP1 gene sequence (nucleotide position 196-447) was performed using the isolates in 2010, 52 previous CA6 strains isolated in Taiwan from 1998 through 2009, along with 8 sequences obtained from Genbank (7 strains from Finland, and the prototype strain, Gdula)**. Clade I included the 52 strains isolated from 1998 through 2009 in Taiwan, while clade II included the 123 strains isolated in Taiwan in 2010 (the compressed subtree indicated by the bold triangle) and the 7 strains isolated in Finland in 2008.

Based on the phylogenetic analysis of the partial VP1 nucleotide sequence, the CA6 strains, except the prototype strain Gdula, were divided into two clades (Figure 2). Clade I was comprised of the 52 strains isolated from 1998 through 2009 in Taiwan, while clade II was comprised of the 123 strains isolated from the Taiwanese patients in 2010 and the 7 strains isolated from Finnish patients in 2008. Within clade II, the VP1 sequences of the isolates from the Taiwanese patients in 2010 were 92.8% to 96.8% identical to those from the Finnish patients in 2008.

## Discussion

The results of his study suggest that the onychomadesis epidemic which occurred in Taiwan was significantly associated with the HFMD caused by the CA6 infection. In addition to onychomadesis, our study found that HFMD patients with CA6 infection presented with more widespread skin lesions and more profound tissue destruction. The CA6 strains circulating in 2010 differed from those circulating before 2010, not only in terms of clinical manifestations, but also in the genetic sequences of the VP1 genes.

The fact that HFMD patients with CA6 infection had more widespread skin lesions also explains the difference in the percentage of nail abnormalities in CA6 and non-CA6 HFMD cases. Expanded skin site involvement in our study indicated a broad spectrum of direct cell infection by the CA6 virus. Onychomadesis is a painless, non-inflammatory nail change that is idiopathic or results from a wide range of systemic diseases or drug exposures [17]. The mechanism of onychomadesis in our study remains unclear. It has been speculated that onychomadesis occurs secondary to inflammation of the nail matrix or maceration associated finger blisters [7]. In the outbreak of hand, foot, and mouth disease (HFMD) which occurred in Finland in 2008, in which onychomadesis was a common symptom, Osterback et al. detected CA6 in shed nail fragments of a patient who had onychomadesis following a HFMD episode by using RT-PCR and suggested that CA6 virus replication damaged the nail matrix, resulting in onychomadesis [8]. Although direct evidence of a viral infection in the nail matrix was not obtained, our study adds to the mounting evidence of a causal relationship between CA6 HFMD and nail abnormalities.

In addition to CA6 infection, we found that some patients with echovirus 30, EV71, and CA16 infection also experienced nail abnormalities. In an onychomadesis outbreak in Spain, Davia et al. found that several serotypes of enteroviruses, such as coxsackieviruses A5, A6, A16, B1, B3, echoviruses 3, 4, and 9, and enterovirus 71, may cause onychomadesis, in addition to the major enterovirus serotype, coxsackievirus A10, and concluded that more than one enterovirus serotype was implicated in the nail matrix arrest outbreak [5]. Our study supports this observation. However, there were no reports of nail abnormalities associated with the aforementioned enterovirus serotypes in Taiwan. Whether or not another enterovirus serotype, other than CA6, is associated with nail changes warrants further investigation.

The validity of virus isolation results was crucial to the study. Specimens obtained from skin blisters were the most appropriate for microbiological studies for HFMD. The majority of the specimens were obtained from pharyngeal swabs and we took the specimens within 3 days of HFMD onset. Although the results of the pharyngeal swabs did not provide direct evidence of a causal relationship, we believe that there is a high degree of association between CA6 and HFMD.

There are several limitations to this study. Recall bias of enrolled patients may exist because of the telephone interview. The gap period between the telephone interview, which was carried out in early 2011, and the HFMD episode may have been as long as 8 months, and the enrolled patients may not have been able to accurately describe the details of onychomadesis or the skin sites involved. In addition, the prevalence of the nail changes may have been underestimated due to patients' unawareness of them. Since the nail changes associated with HFMD are generally painless, the presence of nail changes may not have drawn the attention of the patients.

## Conclusions

In conclusion, the onychomadesis epidemic following the HFMD episodes was primarily associated with an outbreak of CA6 infection in 2010. CA6 infections attacked a broader spectrum of skin sites and caused more profound tissue damage. The VP1 gene sequences of the CA6 strains isolated from Taiwanese patients show a high degree of similarity with those isolated from Finnish patients in 2008, but differ from those previously circulating in Taiwan from 1998 through 2009.

## Consent section

Informed consent for participation in the study was obtained from the patients or their guardians. Consent to publish the images in Figure 1 was obtained from the parents of the patients.

## Competing interests

The authors declare that they have no competing interests.

## Authors' contributions

The first author, Dr. SHW, and the corresponding author, Dr. LYC, designed and conducted this clinical study, and revised this manuscript. Dr. Sung-Hsi Wei, enrolled the cases, performed the analyses, and wrote the manuscript. Dr. Yuan-Pin Huang, Dr. Ming-Chih Liu, Dr. Tsung-Pei Tsou, Dr. Hui-Chen Lin, MS. Tsuey-Li Lin, Dr. Chen-Yen Tsai, Dr. Yen-Nan Chao and Chun-Ming Hsu collected cases and revised the manuscript.

## Pre-publication history

The pre-publication history for this paper can be accessed here:

http://www.biomedcentral.com/1471-2334/11/346/prepub
